# Progress and Developments in the Fabrication and Characterization of Metal Halide Perovskites for Photovoltaic Applications

**DOI:** 10.3390/nano15080613

**Published:** 2025-04-16

**Authors:** Faouzia Tayari, Silvia Soreto Teixeira, Manuel Pedro F. Graca, Kais Iben Nassar

**Affiliations:** I3N-Aveiro, Department of Physics, University of Aveiro, 3810-193 Aveiro, Portugal; faouzia.tayari@ua.pt (F.T.); silvia.soreto@ua.pt (S.S.T.); mpfg@ua.pt (M.P.F.G.)

**Keywords:** metal halide perovskites, photovoltaics, solar cells, fabrication techniques, characterization methods, optoelectronic properties, stability, degradation mechanisms, efficiency, scalability, device integration

## Abstract

Metal halide perovskites have emerged as a groundbreaking material class for photovoltaic applications, owing to their exceptional optoelectronic properties, tunable bandgap, and cost-effective fabrication processes. This review offers a comprehensive analysis of recent advancements in synthesis, structural engineering, and characterization of metal halide perovskites for efficient solar energy conversion. We explore a range of fabrication techniques, including solution processing, vapor deposition, and nanostructuring, emphasizing their impact on material stability, efficiency, and scalability. Additionally, we discuss key characterization methods, such as X-ray diffraction, electron microscopy, impedance spectroscopy, and optical analysis, that provide insights into the structural, electrical, and optical properties of these materials. Despite significant progress, challenges related to long-term stability, degradation mechanisms, and environmental sustainability persist. This review delves into current strategies for enhancing the durability and performance of perovskite-based photovoltaics and highlights emerging trends in device integration and commercialization. Finally, we provide future perspectives on optimizing material design and overcoming existing limitations to guide continued research in this rapidly advancing field.

## 1. Introduction

Perovskites represent a broad class of crystalline materials that share the general formula ABX_3_, where A is a large cation (typically an organic or inorganic monovalent cation such as methylammonium (MA^+^), formamidinium (FA^+^), or cesium (Cs^+^)), B is a smaller metal cation (such as lead (Pb^2^^+^), tin (Sn^2^^+^), or germanium (Ge^2^^+^)), and X is a halide anion (such as chloride (Cl^−^), bromide (Br^−^), or iodide (I^−^)). The structural flexibility and chemical versatility of perovskites have led to their rapid adoption in various energy-related applications, particularly in the field of photovoltaics [1,2,3,4,5,6]. Their high absorption coefficients, long-charge carrier diffusion lengths, and defect-tolerant properties make them excellent candidates for next-generation solar cells. Since the introduction of organic–inorganic halide perovskite solar cells in 2009, their power conversion efficiency (PCE) has skyrocketed from ~3% to over 25%, surpassing many conventional semiconductor-based solar technologies [7,8,9,10,11,12]. The exceptional optoelectronic properties of perovskite materials stem from their unique electronic band structures, which enable efficient light absorption and charge transport. Furthermore, their ability to be synthesized through low-cost solution processing methods provides an economic advantage over silicon-based photovoltaics, paving the way for scalable production [13,14,15,16,17]. However, despite these promising attributes, the commercialization of perovskite solar cells remains hindered by several challenges, including long-term stability issues, sensitivity to environmental factors such as moisture and UV exposure, and concerns regarding lead toxicity. These factors necessitate further research into advanced fabrication methods, robust encapsulation strategies, and the development of lead-free alternatives [18,19,20,21,22]. The fabrication of metal halide perovskites (MHPs) has evolved through a range of synthesis techniques, including solution processing, chemical vapor deposition (CVD), and nanostructuring methods, each offering distinct advantages in terms of material quality, stability, and efficiency. Understanding the impact of synthesis parameters on the final material properties is crucial for optimizing device performance [23,24,25,26,27,28]. Characterization techniques such as X-ray diffraction (XRD), scanning electron microscopy (SEM), transmission electron microscopy (TEM), and impedance spectroscopy play a vital role in elucidating the structural, morphological, and electrical properties of perovskite materials [29,30,31,32,33,34]. In addition to conventional characterization techniques, in situ photoluminescence (PL) has become increasingly popular for monitoring the crystallization process of perovskite materials, providing insights into the evolution of their optical properties during film formation [35,36].

These analytical tools provide insights into crystallinity, grain size, defect states, and charge transport mechanisms, which are essential for designing high-performance perovskite solar cells. Additionally, advances in machine learning and artificial intelligence (AI) are being integrated into material discovery and characterization, allowing for the rapid screening of new perovskite compositions with enhanced properties. As researchers continue to explore innovative ways to improve perovskite solar cells, new frontiers such as tandem architectures, quantum-dot-based perovskites, and flexible photovoltaic devices are gaining attention. These developments promise to expand the application scope of perovskite materials beyond traditional rigid solar panels, enabling their integration into wearable electronics, building-integrated photovoltaics, and other emerging technologies. This review aims to provide a detailed analysis of the recent advancements in perovskite nanomaterial fabrication and characterization, discuss the existing challenges, and highlight future directions for enhancing the performance and durability of perovskite-based photovoltaic devices.

## 2. Characterization Techniques

To evaluate and optimize the structural, optical, electrical, and morphological properties of metal halide perovskites, a variety of advanced characterization techniques were employed. These methods provide essential insights into material quality, stability, and performance, ultimately guiding the development of more efficient perovskite-based photovoltaic devices. Table 1 summarizes all the techniques used [37,38,39,40].

### 2.1. Structural Characterization

**X-ray diffraction (XRD)**: XRD (PANalytical, Aveiro, Portugal) was utilized to analyze the crystallinity, phase purity, and lattice structure of the synthesized metal halide perovskites. By examining diffraction patterns, key parameters such as unit cell dimensions and crystallite size (using the Scherrer equation) were determined. XRD data also provided insights into phase transitions and potential secondary phases that could impact material stability and performance in photovoltaic applications [40,41,42,43,44,45,46,47,48]. For example, improved crystallinity achieved through optimized processing methods often correlates with enhanced charge transport properties, crucial for efficient energy conversion in solar cells.

**Raman spectroscopy**: (Raman-SNOM, Aveiro, Portugal), provided detailed information about the vibrational modes, lattice dynamics, and structural stability of the perovskite materials. This technique is particularly valuable for identifying stress-induced changes, organic-inorganic interactions, and phase transitions, which can directly affect material stability under operational conditions [49,50,51,52,53,54,55,56,57]. Raman shifts were used to track bonding environments, revealing how specific synthesis routes or postprocessing treatments can influence material quality and performance.

### 2.2. Morphological Analysis

**Scanning electron microscopy (SEM)**: SEM (performed at the Electron Microscopy Service of the University of Aveiro—SME-UA, Aveiro, Portugal), was employed to investigate the surface morphology, grain size distribution, and uniformity of perovskite films. This technique was critical for assessing how different synthesis methods (such as spin coating or vapor deposition) impacted film quality and the formation of defects [58,59,60,61,62,63,64,65]. The secondary electron images provided insights into surface roughness, while backscattered electron imaging allowed for the identification of compositional variations across the films, which can influence charge transport and overall device performance.

**Transmission electron microscopy (TEM)**: TEM analyses were conducted at the Institute of Nanostructures, Nanomodelling, and Nanofabrication at Aveiro, Portugal, utilizing a JEOL 2200FS and a Hitachi HD2700 STEM (non-magnetic samples). TEM provided high-resolution images of nanocrystals, grain boundaries, and interfaces within the perovskite films. Using high-angle annular dark field (HAADF) imaging and energy-dispersive X-ray spectroscopy (EDS), elemental distribution and crystal structure were analyzed. Selected area electron diffraction (SAED) patterns confirmed crystallinity, while high-resolution TEM (HRTEM) revealed atomic-level structural details, allowing researchers to better understand how defect structures or grain boundaries affect the material’s photovoltaic properties [30,66,67,68,69,70,71,72,73,74,75,76,77,78,79,80,81,82].

### 2.3. Optical Characterization

**Ultraviolet–visible (UV–Vis) spectroscopy**: UV-Vis absorption spectroscopy (Spectrometer UV/Vis, Aveiro, Portugal) was employed to measure the optical absorption characteristics and bandgap energy of metal halide perovskites. Tauc plots were used to extract the optical bandgap, which is a key factor for optimizing light absorption in photovoltaic devices [83,84,85,86,87,88,89]. Variations in absorption spectra provided valuable information about the quality of the films, crystallization processes, and the presence of defect states that could hinder the photovoltaic performance.

**Photoluminescence (PL) spectroscopy**: PL spectroscopy was used to study charge carrier recombination dynamics, defect states, and excitonic behavior in metal halide perovskites [89,90,91,92,93,94,95,96,97,98,99,100]. These measurements were conducted at the Optical Spectroscopy at Aveiro, Portugal. Time-resolved PL (TRPL) measurements further helped determine carrier lifetime and nonradiative recombination processes, both of which play a significant role in the efficiency of perovskite solar cells. Optimizing these parameters through careful synthesis and processing can enhance the material’s ability to convert light into electricity.

**Fourier-transform infrared spectroscopy (FTIR)**: FTIR (Optical Spectroscopy Lab, i3N, University of Aveiro, Portugal) was applied to analyze molecular interactions and functional group vibrations in hybrid perovskites [101,102,103]. This technique is particularly useful for detecting organic–inorganic interactions, moisture-related degradation, and other factors that could affect the material’s long-term stability and efficiency in photovoltaic applications.

### 2.4. Electrical and Dielectric Measurements

**Impedance spectroscopy**: IS (i3N, University of Aveiro, Portugal) was used to analyze charge transport properties, resistivity, dielectric relaxation, and interfacial polarization effects in metal halide perovskites. By interpreting Nyquist plots and employing equivalent circuit modeling, key parameters such as charge transfer resistance, grain boundary effects, and ion migration were assessed [104,105]. These measurements are critical for optimizing the electrical performance of perovskite solar cells.

**Hall Effect measurements**: (HEMS Aveiro, Portugal) effect analysis provided essential data on charge carrier concentration, mobility, and type (n-type or p-type), helping to optimize transport properties for photovoltaic applications. These measurements are directly related to how well the material can conduct electricity when exposed to light, which is a fundamental aspect of solar cell performance [106,107,108].

**Current–voltage (I–V) measurements**: I–V characterization was performed under both dark and illuminated conditions to evaluate the photovoltaic performance of perovskite devices (I–V, Aveiro, Portugal). Key parameters, such as short-circuit current density (Jsc), open-circuit voltage (Voc), and fill factor (FF), were measured to assess overall device efficiency [109,110,111]. These measurements helped determine how various material synthesis strategies influenced photovoltaic output.

**Thermally stimulated current (TSC) measurements**: TSC measurements were conducted to investigate trap states (TSC, Aveiro, Portugal), charge carrier detraining mechanisms, and temperature-dependent charge transport in perovskite films [112]. This technique provides insight into charge carrier behavior under different thermal conditions, aiding in the development of more stable perovskite-based photovoltaic materials.

### 2.5. Integration of Emerging Techniques

The integration of machine learning (ML) and artificial intelligence (AI) into the characterization of metal halide perovskites is an emerging trend that promises to accelerate material optimization. By analyzing vast datasets generated from characterization techniques such as XRD, PL, and I–V measurements, ML models can predict material properties and suggest optimal synthesis conditions [113]. This AI-driven approach can significantly reduce the time required to discover and optimize new perovskite compositions, leading to more efficient and stable photovoltaic materials.

## 3. Synthesis Methods for Metal Halide Perovskites

The synthesis of metal halide perovskites is a key factor in defining their crystal structure, grain size, surface morphology, and optoelectronic properties, all of which impact their performance in energy-related applications. Different synthesis methods have been explored to optimize material stability, defect control, and process scalability. These methods generally fall into three categories.

### 3.1. Solution-Based Synthesis

Solution-based methods are widely used for perovskite synthesis due to their simplicity, scalability, and cost-effectiveness. These techniques involve dissolving precursor materials in solvents to promote chemical reactions that lead to perovskite crystallization. They are particularly suitable for producing thin films and bulk materials with controlled stoichiometry. However, solution-based methods can lead to defects if the process is not well controlled, which can negatively affect the final material quality.

#### 3.1.1. Sol–Gel Method

The sol–gel method is a chemical synthesis route where metal precursors undergo hydrolysis and condensation to form a colloidal solution (sol). The sol gradually transforms into a gel, which, upon drying and annealing, forms the crystalline perovskite structure. This method is valued for its ability to provide fine control over material stoichiometry and purity, leading to highly uniform and nanostructured films [114]. It works well for both thin films and bulk materials, making it versatile for various applications. However, the sol–gel method requires high-temperature post-annealing to improve crystallinity, and it is sensitive to moisture and precursor quality, which can impact film uniformity.

#### 3.1.2. Spin-Coating and Dip-Coating

Spin-coating and dip-coating are common liquid deposition techniques used for perovskite film formation. In spin-coating, the precursor solution is dropped onto a rotating substrate, causing the solution to spread evenly due to centrifugal force. This method is widely used for thin-film deposition in perovskite solar cells [115]. In dip-coating, a substrate is dipped into the precursor solution and then slowly withdrawn, allowing the film to form as the solvent evaporates. Dip-coating is useful for producing thicker films but requires precise control of the withdrawal speed to ensure uniformity. Both methods can lead to films with pinholes or roughness if the process is not optimized, and they are not ideal for large-area coatings due to thickness variations.

#### 3.1.3. Ligand-Assisted Reprecipitation (LARP)

Ligand-assisted reprecipitation (LARP) is a technique used to synthesize monodisperse perovskite nanocrystals at room temperature. By carefully controlling the concentration of organic ligands (such as oleic acid and amines), the nucleation and growth of perovskite nanoparticles can be precisely tuned. This method is particularly beneficial for producing perovskite quantum dots (PQDs) with high luminescence and controlled bandgap tuning. It is ideal for applications in light-emitting diodes (LEDs) and photodetectors [116]. However, LARP requires efficient surface passivation to prevent degradation, and the perovskite nanocrystals may face stability issues when exposed to air and moisture.

### 3.2. Vapor-Phase Deposition Techniques

Vapor-phase deposition techniques offer improved film quality, better stability, and controlled morphology, making them highly suitable for high-performance devices. These methods generally involve the deposition of perovskite precursors in the vapor phase, leading to high-quality films with fewer defects compared to solution-based methods [117]. However, vapor-phase deposition techniques require specialized equipment and are more complex and expensive than solution-based methods.

#### 3.2.1. Thermal Evaporation

In thermal evaporation, perovskite precursors are heated in a vacuum, causing them to sublimate and deposit as a thin film on a substrate. This technique is solvent-free, which reduces the likelihood of defects in the final film. Thermal evaporation can produce dense, uniform films with minimal impurities, making it suitable for multilayer device fabrication [118]. However, this method requires expensive vacuum systems and is limited to materials with suitable vapor pressures, which can restrict the range of materials that can be processed.

#### 3.2.2. Chemical Vapor Deposition (CVD)

Chemical vapor deposition (CVD) involves the reaction of gaseous precursors on a heated substrate, which leads to the growth of a thin film. Variants of CVD, such as vapor-assisted CVD (VACVD) and plasma-enhanced CVD (PECVD), offer additional control over film quality and morphology. CVD techniques enable excellent thickness control and produce highly crystalline perovskites with superior electronic properties [119]. However, they are associated with high processing costs, complex instrumentation, and sensitivity to reaction conditions such as temperature and pressure.

### 3.3. Nanostructuring Approaches

Nanostructuring approaches are used to manipulate the properties of perovskite materials by precisely controlling their dimensions at the nanoscale. These methods enhance material functionality by improving light absorption, charge transport, and mechanical stability. Nanostructured perovskites are crucial for improving the performance of optoelectronic devices such as solar cells and LEDs [120,121,122]. However, nanostructuring requires advanced fabrication techniques, which can be challenging to scale up.

#### 3.3.1. Colloidal Synthesis

**Figure 1:**
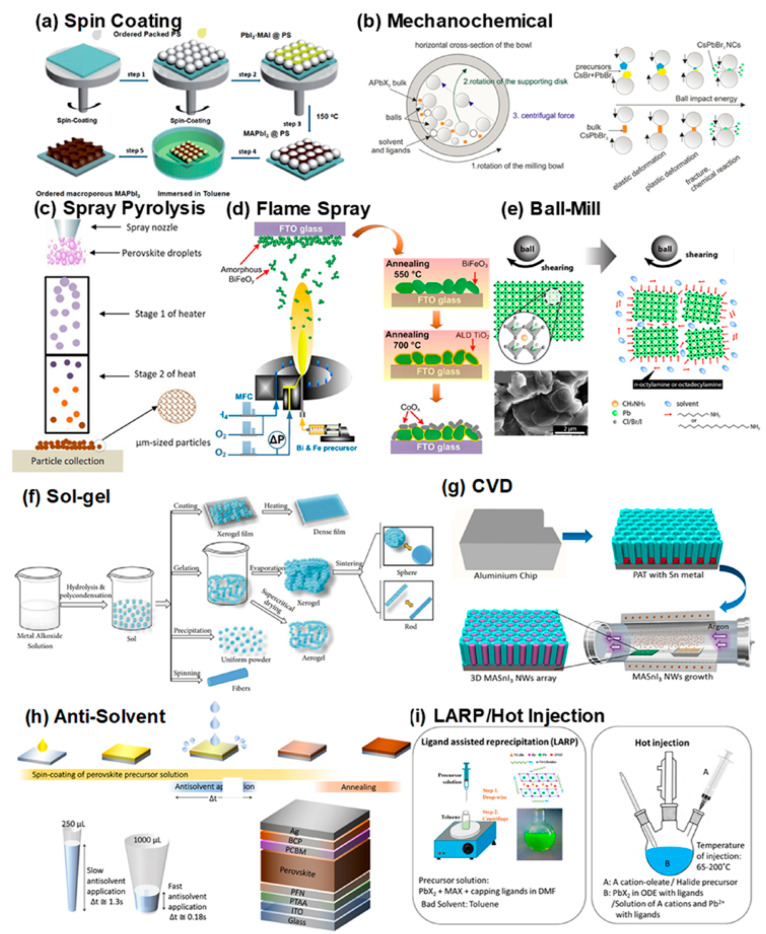
Different techniques for synthesizing perovskite and nanocomposite structures: (**a**) spin coating, (**b**) mechanochemical synthesis, (**c**) spray pyrolysis, (**d**) flame spray pyrolysis, (**e**) ball milling, (**f**) sol–gel method, (**g**) chemical vapor deposition (CVD), (**h**) antisolvent approach, and (**i**) ligand-assisted reprecipitation (LARP) with hot injection [123].

Colloidal synthesis involves precipitating perovskite nanoparticles in a solvent and stabilizing them with organic ligands. This approach enables the production of highly luminescent, defect-tolerant nanocrystals with tenable optoelectronic properties. Colloidal perovskite nanocrystals are valuable for applications such as light-emitting diodes (LEDs) and photodetectors. However, colloidal synthesis requires careful surface engineering to prevent degradation and ensure long-term stability. Figure 1 describes all the techniques for synthesizing perovskite and nanocomposite structures [123].

#### 3.3.2. Template-Assisted Growth

Template-assisted growth uses prepatterned nanostructures to guide perovskite crystallization, resulting in well-ordered nanowires, nanotubes, and mesoporous structures. This approach can improve charge transport by creating structured pathways for charge carriers, which, in turn, enhances device efficiency. However, template-assisted growth requires precise fabrication techniques to achieve the desired nanostructures, and the process can be complex and time-consuming.

### 3.4. Comparative Analysis of Synthesis Methods

To evaluate the different perovskite synthesis techniques, a comparative analysis of their advantages, limitations, and application areas is provided below. This table highlights key characteristics of each method, offering insights into their suitability for various applications, while also pointing out the challenges that need to be addressed for optimal performance. Table 2 summarizes the comparative analysis of perovskite synthesis methods.

## 4. Optimization of Metal Halide Perovskites for Photovoltaics

**Figure 2:**
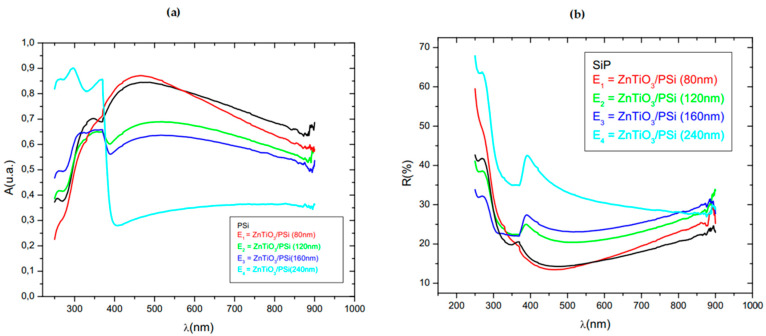
UV-Vis absorbance and reflectivity spectra of ZnTiO_3_ nanostructures with different thicknesses grown on porous silicon substrate [124].

Marwen et al. [124] investigated the morphological, structural, and optical properties of ZnTiO_3_ nanostructures deposited on silicon and porous silicon substrates using the sol–gel method. Their study revealed that substrate porosity plays a crucial role in determining the crystallite size, optical absorption, and surface morphology of ZnTiO_3_ layers. XRD analysis confirmed a decrease in crystalline size for ZnTiO_3_ films grown on porous silicon, with an average grain size of approximately 80 nm. Figure 2 presents the UV-Vis absorbance and reflectivity spectra of ZnTiO_3_ nanostructures with different thicknesses deposited on PSi. The results indicate that ZnTiO_3_/PSi structures exhibit enhanced photon absorption in the UV region (250–400 nm), attributed to Surface Plasmon Resonance (SPR) effects and improved light trapping within the porous network. However, increasing the ZnTiO_3_ layer thickness resulted in a decrease in visible-light absorption due to film smoothing and grain growth. Reflectance measurements showed that ZnTiO_3_ deposition reduced surface reflectivity at specific wavelengths, further enhancing light absorption. These findings highlight the potential of ZnTiO_3_/PSi nanostructures for improving light-harvesting efficiency in photovoltaic and optoelectronic applications.

**Figure 3:**
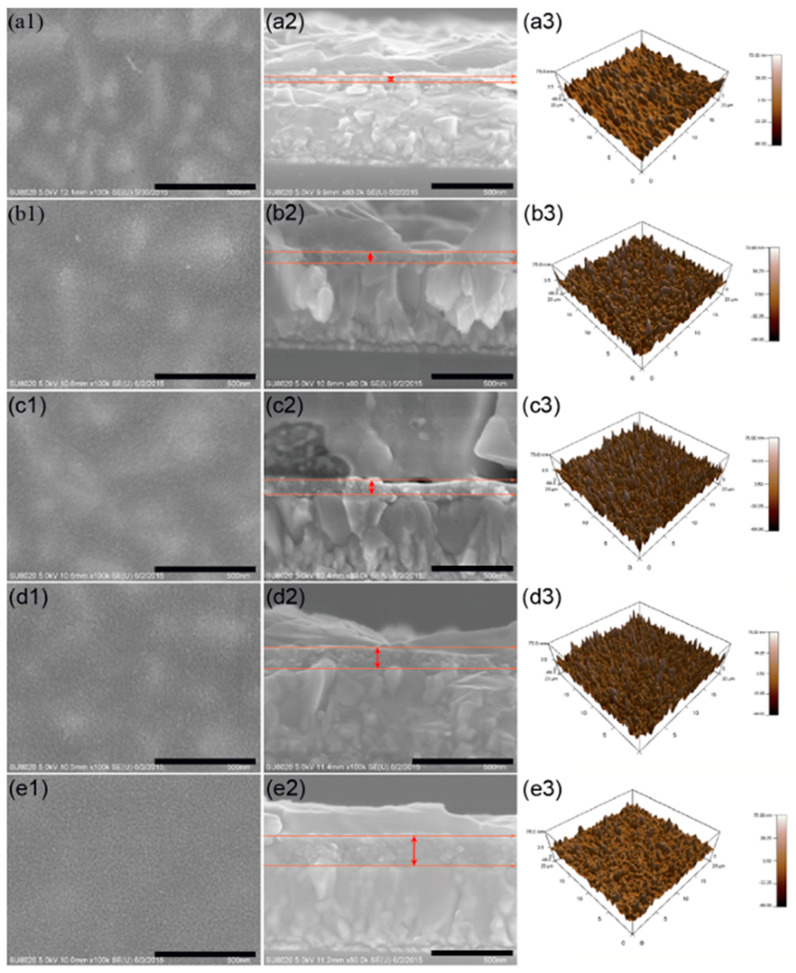
SEM and AFM images of TiO_2_ compact layers prepared from sols aged for different durations, along with UV-Vis absorption spectra of perovskite films deposited on these layers [125].

**Figure 4:**
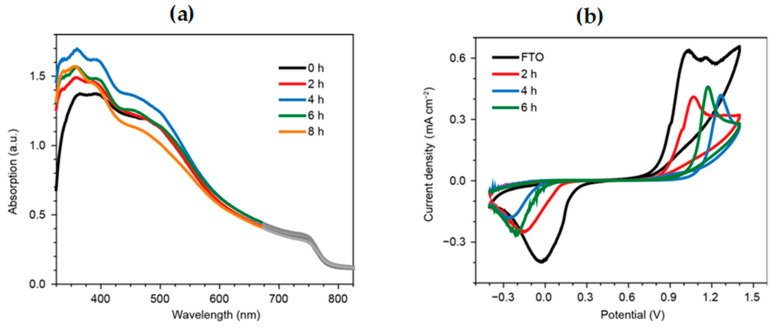
Cyclic voltammetry (CV) and electrochemical impedance spectroscopy (EIS) measurements of perovskite solar cells with different TiO_2_ compact layers, highlighting the effect of sol aging time on charge blocking and recombination properties [125].

Guo et al. [125] investigated the influence of TiO_2_ compact layer morphology on perovskite solar cell performance by analyzing surface characteristics, optical absorption, charge transfer, and recombination properties. Figure 3 presents SEM and AFM images of TiO_2_ layers from sols aged for different durations, along with UV-Vis absorption spectra of perovskite films deposited on these layers. The 4 h aged sol produces the most uniform and compact TiO_2_ film, improving perovskite adhesion and coverage. UV-Vis spectra show that perovskite films deposited on this layer exhibit the highest absorption due to enhanced surface roughness, which increases light scattering and optical path length. In contrast, shorter or longer aging times lead to poor surface coverage or excessive roughness, reducing light absorption. Figure 4 combines cyclic voltammetry (CV) and electrochemical impedance spectroscopy (EIS) to evaluate charge blocking and transfer properties. CV results indicate that perovskite solar cells without a TiO_2_ compact layer suffer from severe charge recombination, with a small separation voltage (~1.1 V) and high peak current density (~0.63 mA cm^−2^). The inclusion of a TiO_2_ compact layer enhances charge blocking, with the 4 h aged sol-derived layer achieving the highest separation voltage (~1.27 V). However, excessive aging (6 h) leads to crack formation, reducing separation voltage (~1.17 V) and facilitating charge leakage. EIS results support this by showing that the 4 h aged TiO_2_ layer has the highest charge transfer resistance, indicating strong suppression of charge recombination. In contrast, the absence of a compact layer or an over-aged (6 h) TiO_2_ layer results in lower charge transfer resistance, leading to efficiency loss.

Yong Chen et al. [126] have demonstrated the MHPs for both photovoltaic energy conversion and solar-to-fuel applications, such as water splitting and CO_2_ reduction. Building on these insights, this review explores recent advancements in synthesis, structural engineering, and characterization of MHPs for efficient solar energy conversion. Various fabrication methods, including solution processing, vapor deposition, and nanostructuring, are examined, emphasizing their impact on material stability, efficiency, and scalability. Key characterization techniques, such as X-ray diffraction, electron microscopy, and impedance spectroscopy, are discussed to provide a deeper understanding of their structural and optoelectronic properties. Despite significant progress, challenges related to long-term stability, environmental impact, and degradation mechanisms persist. This review highlights emerging strategies to enhance the durability and performance of MHP-based photovoltaics and solar-to-fuel systems, while also addressing opportunities for future commercialization and integration into energy storage technologies.

**Figure 5:**
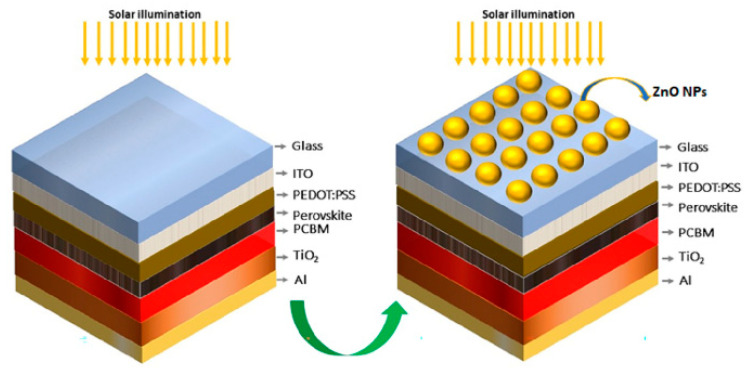
Setup for electrical experiments with perovskite device and ZnO NPs/perovskite device [127].

In the study by Ricardo et al. [127], the performance of perovskite solar cells (PSCs) was enhanced by employing zinc oxide (ZnO) nanoparticles (NPs) as nanodiffusers. The ZnO NPs, with an average size of 160 nm, were carefully deposited on the surface of the perovskite solar cells, which led to significant improvements in device performance. These nanoparticles reduce reflection and increase the absorption of solar radiation in the photovoltaic active layer by enhancing light trapping and scattering. The study utilized both experimental and computational simulations to analyze the effects of ZnO NPs on the solar cells’ efficiency. The results from optical computational modeling revealed that the ZnO nanospheres scattered solar radiation predominantly in the forward direction, reducing device reflectance. This efficient light coupling into the active layer improved photocurrent generation and resulted in a 23.5% increase in photovoltaic device efficiency, from 10.6% to 13.1%. The experimental setup for the electrical evaluation of perovskite cells, with and without the ZnO NP coating, is shown in Figure 5, which illustrates the current density versus voltage characteristics of the devices. This novel approach demonstrates how the integration of ZnO nanoparticles can enhance the efficiency of perovskite solar cells by improving their optical and electrical properties.

**Figure 6:**
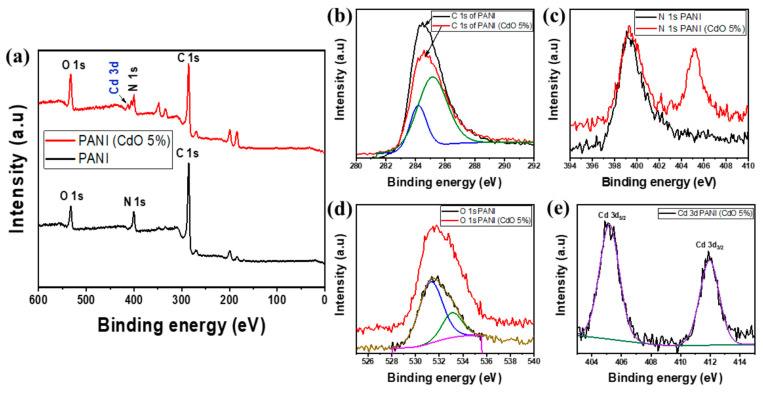
(**a**) XPS survey for a pure PANI and PANI (CdO 5%); (**b**) C 1s (**c**) N 1s; (**d**) O 1s; and (**e**) Cd 3d for pure PANI and PANI (CdO 5%) [128].

In the study by Ashraf et al. [128], the authors explore the synthesis, characterization, and electrical properties of CdO-doped polyaniline (PANI) as a hole transport layer (HTL) in inverted perovskite solar cells (PSCs). The goal of doping PANI with cadmium oxide (CdO) was to enhance the hole extraction properties and overall performance of the PSCs. The researchers synthesized PANI doped with varying amounts of CdO (0%, 1%, 5%, and 10%) and characterized the samples using a range of techniques, including X-ray diffraction (XRD), X-ray photoelectron spectroscopy (XPS), Fourier transform infrared spectroscopy (FTIR), Raman spectroscopy, scanning electron microscopy (SEM), and transmission electron microscopy (TEM). Electrical measurements revealed that the addition of CdO significantly improved the conductivity and mobility of PANI, leading to better device performance. Specifically, the PSC with 5% CdO-doped PANI exhibited a power conversion efficiency (PCE) of 14.71%, compared to 13.38% for the device using pristine PEDOT:PSS. Moreover, the open-circuit voltage (V_OC) improved from 1.02 V for the PEDOT:PSS device to 1.11 V for the CdO-doped PANI device, indicating better energy band alignment and reduced recombination losses. Additionally, the short-circuit current density (J_SC) increased from 17.01 mA/cm^2^ to 17.67 mA/cm^2^, reflecting enhanced charge extraction and transport efficiency. Figure 6 presents the XPS results for pure PANI and PANI doped with 5% CdO. The XPS spectra confirm the incorporation of CdO into the PANI matrix, as evidenced by the presence of Cd 3d peaks alongside the typical peaks for C 1s, N 1s, and O 1s. The results suggest that the doping of PANI with CdO leads to significant changes in the electronic environment, enhancing the overall performance of the inverted PSCs. This study highlights the potential of CdO-doped PANI as an effective HTL material for improving the performance of perovskite solar cells [128].

## 5. Machine Learning and AI in Perovskite Research

The integration of machine learning (ML) and artificial intelligence (AI) in perovskite solar cell (PSC) research has emerged as a powerful tool for accelerating material discovery, optimizing fabrication processes, and improving device performance. Traditional experimental approaches for screening perovskite compositions and processing conditions are time-consuming and require extensive resources. In contrast, ML techniques enable high-throughput screening, predictive modeling, and automated data analysis, significantly reducing the trial-and-error aspect of material development. Machine learning has also been employed to refine fabrication conditions for PSCs. Factors such as annealing temperature, precursor concentration, and solvent engineering significantly influence the crystallization of perovskite films. Seongtak et al. [129] developed an ML defect analysis and optimization for P-I-N-structured perovskite solar cells. Such AI-driven process optimization ensures higher reproducibility and scalability of perovskite-based devices.

**Figure 7:**
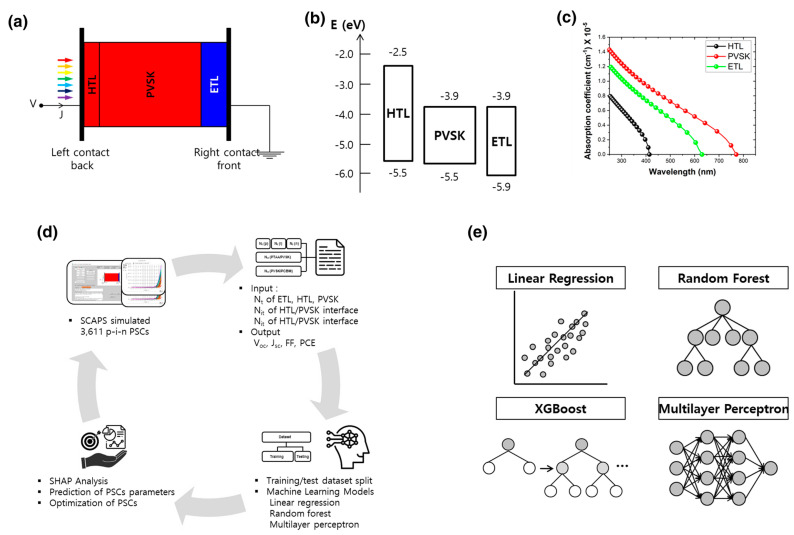
(**a**) PSC architecture for SCAPS; (**b**) energy band diagram of P-I-N-structured PSCs; (**c**) absorption coefficients of HTL, PVSK, and ETL as input parameters for SCAPS; (**d**) overall process schematic for defect analysis and optimization of PSCs using ML training and prediction; and (**e**) the four ML algorithms used for training, test, and prediction [129].

They [129] conducted a study on machine-learning-assisted defect analysis and optimization for P-I-N-structured perovskite solar cells. Using the SCAPS-1D solar cell capacitance simulator, they generated 3611 datasets with varying defect densities in both the bulk and interfaces of PSCs. Four different machine learning models were trained, with the random forest algorithm achieving the highest accuracy (0.999) and lowest root mean square error (0.00306). The study employed Shapley Additive Explanations (SHAP) analysis to determine which defects had the most significant impact on power conversion efficiency (PCE). The results highlighted that bulk defects within the perovskite layer and interfacial defects at the hole transport layer (HTL)/perovskite and perovskite/electron transport layer (ETL) junctions played a crucial role in performance degradation. Notably, when the perovskite bulk defect density was low, the sensitivity to interface defect densities increased, indicating a need for precise interface engineering. By leveraging machine-learning-based optimization, the researchers proposed defect management strategies, improving the PCE from 17.97% to 24.66% in poly(triarylamine) (PTAA)/perovskite/phenyl-C61-butyric acid methyl ester (PCBM) solar cells. This methodology not only provides insights into defect-related losses but also offers a powerful tool for manufacturing process optimization. Figure 7 illustrates the P-I-N-structured perovskite solar cell model simulated in SCAPS, along with the input parameters for different layers, including HTL, perovskite absorber, and ETL. The simulation was performed under AM 1.5G solar illumination (100 mW cm^2^) to extract current–voltage (I–V) characteristics. Five types of defect densities were classified: bulk defects in HTL, perovskite, and ETL, as well as interfacial defects at HTL/perovskite and perovskite/ETL junctions. The dataset generated through SCAPS served as the training set for machine learning models to predict and optimize solar cell efficiency. This study demonstrates the potential of AI-driven optimization in perovskite solar cell design, paving the way for improved device stability and commercial viability. Similar approaches have been applied in other works, such as efficiency prediction for KSnI_3_ PSCs using supervised learning and bandgap prediction of hybrid perovskites with machine learning techniques.

**Figure 8:**
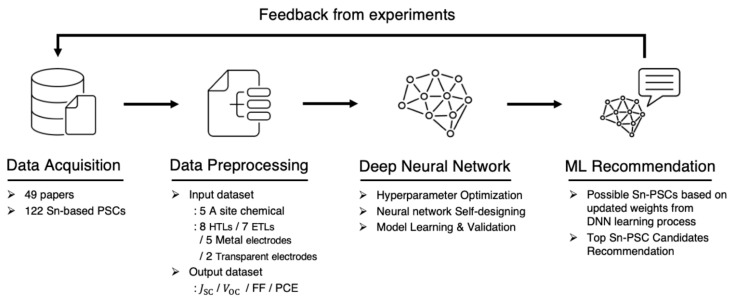
Design recommendation framework of novel Sn PSCs [130].

Taeju Bak et al. [130] explored the use of machine learning (ML) to accelerate the design of lead-free tin (Sn) perovskite solar cells (PSCs), which are considered a promising alternative to lead (Pb) PSCs due to environmental concerns. However, Sn-based PSCs remain in their early development stages and require extensive optimization to achieve high efficiency. Traditional trial-and-error methods are time-consuming and inefficient, prompting the need for AI-driven approaches to streamline device design. To address this, the authors developed a K-fold cross-validation-based deep neural network (DNN) model to maximize the prediction accuracy of Sn PSC performance using a limited experimental dataset. The training dataset consisted of 49 published studies, covering 122 different Sn PSC configurations, with input variables including A-site cation compositions, metal electrodes, transparent electrodes, hole transport layers (HTL), and electron transport layers (ETL). The output parameters focused on key photovoltaic performance metrics such as short-circuit current density (JSC), open-circuit voltage (VOC), and fill factor (FF). Figure 8 outlines the four-stage ML framework for Sn PSC design: (i) data collection—extracting experimental data from literature sources, (ii) feature selection and model optimization—identifying critical input variables and optimizing ML model hyperparameters using K-fold cross-validation, (iii) training and validation—improving model accuracy through iterative learning cycles, and (iv) ML-based recommendations—predicting high-performance Sn PSC structures for fabrication and testing. A validation experiment demonstrated that ML-designed Sn PSCs achieved a threefold increase in efficiency (5.57%) compared to unguided fabrication methods (1.72%), highlighting the potential of AI-driven optimization in perovskite research. These findings reinforce the growing role of machine learning in material discovery, defect engineering, and device architecture optimization. AI-based approaches have been successfully applied in other studies, such as SCAPS-based defect analysis for Pb PSCs and bandgap prediction of hybrid perovskites, further demonstrating how computational techniques can accelerate solar cell development while reducing experimental costs [131].

**Figure 9:**
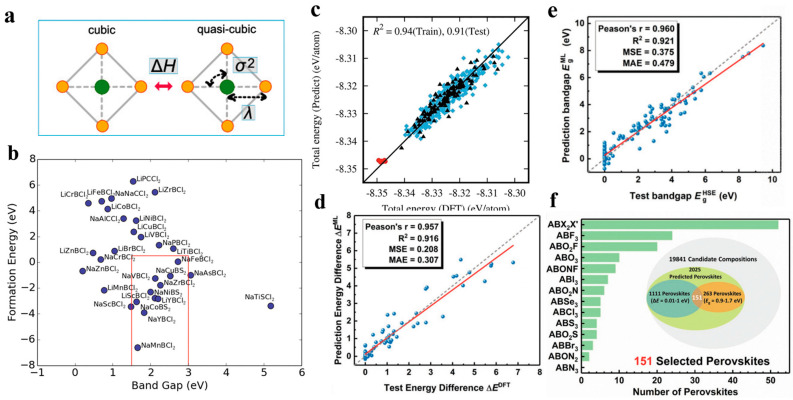
Various ML-based analyses of perovskite materials. (**a**) The parameters ΔH, λ, and σ^2^ were employed to assess deviations from the ideal cubic perovskite structure; (**b**) The formation energy and bandgap (Eg) of Li(Na)BX_3_ perovskites were predicted using DFT, with the materials inside the red-marked region exhibiting optimal values; (**c**) A comparison between the total energy values of BaNbO_2_N supercells in the training (rhombuses) and test sets (triangles) was made, showing strong agreement between ridge regression (RR) predictions and DFT results; (**d**) The predicted phase-transition energy difference (ΔE) was evaluated against DFT calculations, demonstrating high predictive accuracy; (**e**) A comparison between the test bandgap (E_HSEg) and the ML-predicted bandgap (E_MLg) further confirmed the effectiveness of the model; (**f**) A total of 151 promising perovskites with diverse X-site compositions were identified through the ML screening process [131].

Qiuling Tao et al. [131] provided a comprehensive review of (ML) applications in perovskite material design and discovery, highlighting its role in accelerating material development compared to traditional experimental methods. The study outlined key trends in ML-assisted perovskite research, introduced a workflow for ML-based materials discovery, and examined ML applications in predicting and optimizing the properties of inorganic, hybrid organic–inorganic, and double perovskites. Recent advancements include ML-driven stability assessments, such as the work by Park et al., who developed ML models to predict octahedral deformation parameters (ΔHc, λ, and σ^2^) in perovskite structures (Figure 9a in the original paper, referred to as Figure 9 in this review). Additionally, Takahashi et al. used random forest (RF) algorithms to predict the bandgap (E_g_) of ABX_3_ perovskites, identifying 9328 promising candidates for solar cell applications from a dataset of 414,736 compositions, with further density functional theory (DFT) validation confirming the suitability of 11 undiscovered Li/Na-based perovskites (Figure 9b). Another notable study by Kaneko et al. used ML regression models combined with DFT calculations to predict anion ordering stability in BaNbO_2_N supercells, achieving 94% accuracy (Figure 9c). Furthermore, Lu et al. developed a multi-step ML-DFT screening approach to identify stable ferroelectric photovoltaic (FPV) perovskites with high spontaneous polarization and optimal E_g_, successfully extracting 151 candidate materials from a dataset of 19,841 compositions (Figure 9d–f). These studies demonstrate the growing importance of ML in accelerating perovskite material discovery, optimizing structural stability, electronic properties, and energy conversion efficiency, and expanding the database of functional perovskite materials for energy applications [131].

## 6. Performance Metrics of Perovskite Solar Cells

In the Performance Metrics of Perovskite Solar Cells section, it is crucial to focus on the key indicators that are used to evaluate the efficiency and overall performance of perovskite solar cells (PSCs). These metrics are essential for understanding the potential of PSCs in comparison to other photovoltaic technologies and for driving further optimization. Let us break down and interpret the major performance metrics: power conversion efficiency (PCE) is the most significant metric, representing the ratio of the electrical power output (measured in watts) to the total solar power input [132,133,134,135,136,137]. It is typically expressed as a percentage. A higher PCE indicates better solar energy conversion efficiency. In recent years, perovskite solar cells have shown remarkable progress, with efficiencies exceeding 25%, rivaling that of silicon-based solar cells. Researchers continue to improve PCE by optimizing the perovskite layer, electrode materials, and interface engineering. The increase in efficiency can be attributed to advances in material composition (e.g., mixed cations and halides) and device architecture (e.g., tandem cells) [138,139,140,141]. As the performance of PSCs improves, they are becoming more competitive in the market, potentially surpassing traditional solar technologies. Open-circuit voltage (VOC) refers to the maximum voltage available from a solar cell when there is no current flowing through the device. It is a critical indicator of the electrical potential of the cell. A higher VOC indicates better energy harvesting from the sunlight. In perovskite solar cells, improving VOC is an area of ongoing research. Typically, VOC values for PSCs can reach values between 1.1 and 1.2 V, depending on the material and device structure. Achieving higher VOC values is linked to reducing defects and enhancing the charge transport and recombination processes within the material. Researchers are exploring new perovskite compositions to increase VOC without compromising other performance metrics. Short-circuit current density (JSC) is the current generated by the solar cell under illumination at zero voltage. It is a measure of how much current the device can produce under standard test conditions. Higher JSC values indicate better light absorption and charge collection efficiency. Perovskite materials often exhibit high JSC due to their excellent light absorption properties and tunable bandgaps. Typically, JSC values range between 20 and 25 mA/cm^2^ for high-performance devices.

The challenge is to balance JSC with other parameters like VOC and fill factor (FF) to achieve optimal efficiency. Optimization of the perovskite layer thickness, light management techniques (e.g., light trapping), and interface engineering can help enhance JSC. Fill factor (FF) is a measure of the “squareness” of the current–voltage (I–V) curve and indicates how efficiently the solar cell can convert the voltage and current generated under illumination into usable power. The FF is affected by series and parallel resistances within the device, with higher values signifying less energy loss during power conversion. A typical FF for high-efficiency PSCs is around 80–85%, but values can be lower in devices with poor contact or interface issues. Maximizing FF is essential to improve overall device performance [142]. This is achieved by improving the quality of the materials, reducing defects, optimizing the contacts, and ensuring minimal recombination losses. Stability is a critical performance metric for commercializing PSCs. It refers to how well the solar cell maintains its efficiency over time under various environmental conditions (e.g., moisture, temperature, and light exposure). Stability has been one of the biggest challenges for perovskite solar cells, as they are often prone to degradation due to moisture, oxygen, heat, and UV exposure. However, advances in encapsulation techniques, stability testing, and composition engineering have significantly improved the longevity of PSCs. Stability is now a central focus in PSC research, with the aim to match or exceed the lifespan of silicon solar cells (25 years) [143]. A perovskite solar cell with high stability and good performance over time will be crucial for its widespread adoption. Hysteresis refers to the difference in the current–voltage curve during the forward and reverse voltage scans. It can lead to inaccuracies in efficiency measurements and hinder reproducibility. Hysteresis is commonly observed in PSCs due to charge accumulation and interface effects. Reducing hysteresis is essential for improving device stability and obtaining accurate performance measurements. Research is focused on minimizing hysteresis through optimized device architectures, passivation of defects, and the use of interfacial layers to prevent charge trapping. Light soaking refers to exposing a solar cell to continuous light to observe how its performance changes over time. Reverse bias stress tests the ability of the device to withstand reverse voltage without degradation. Light-soaking tests help evaluate the long-term performance and stability under realistic conditions, while reverse bias stress assesses the material’s resilience to electrical stress [144]. These tests are important for ensuring that PSCs can maintain high efficiency in real-world applications. Improvements in the materials and interface layers are crucial for enhancing light-soaking stability and mitigating reverse bias-induced degradation. Photovoltaic yield refers to the amount of energy produced per unit area of the solar cell under standard test conditions. This is an important practical metric, as it directly influences the cost-effectiveness of the technology in large-scale solar installations [145]. A higher photovoltaic yield per unit area makes PSCs more attractive for commercial use. The photovoltaic yield is influenced by factors like light absorption efficiency, charge transport properties, and device design. Maximizing yield is a key goal in the optimization of perovskite solar cells. In summary, the performance metrics of perovskite solar cells, including PCE, VOC, JSC, FF, stability, and hysteresis, are key to understanding and improving the overall efficiency and practicality of these devices. While perovskites show promising performance and efficiency compared to traditional photovoltaic technologies, challenges such as stability and hysteresis remain areas of active research. The continued optimization of materials, device structures, and interface engineering, along with advancements in machine learning and data-driven design, will be critical to overcoming these barriers and making perovskite solar cells commercially viable [146].

## 7. Applications Beyond Photovoltaics

The applications beyond photovoltaics section of perovskite materials are crucial for expanding the understanding of their potential in diverse technological fields. While perovskite solar cells (PSCs) have garnered significant attention due to their high efficiency and low-cost potential for solar energy conversion, their applications extend far beyond photovoltaics. One of the most exciting areas where perovskites are making a significant impact is in light-emitting devices, particularly in perovskite light-emitting diodes (LEDs). Perovskite LEDs have shown impressive performance in terms of high luminescence efficiency, tunable color emission, and ease of fabrication [147,148,149]. This makes them a strong candidate for future applications in displays, lighting, and optical communication technologies. Their remarkable optoelectronic properties, such as high photoluminescence quantum efficiency and long carrier diffusion lengths, make them ideal for efficient light emission. Researchers are exploring ways to optimize perovskite LEDs for high stability and efficiency, working on issues like moisture stability and enhancing the performance of perovskite emitters through material composition and device architecture improvements. In addition to light emission, perovskite materials are being investigated for photodetector applications, where their excellent light absorption properties and fast charge transport capabilities make them suitable for use in high-performance photodetectors. Perovskite photodetectors offer advantages over traditional materials like silicon, including enhanced sensitivity and lower fabrication costs. These devices can be used in a variety of applications, such as environmental monitoring, biomedical imaging, and optical communication systems [150]. Perovskites’ potential for broad absorption spectrum, fast response times, and high signal-to-noise ratios positions them as promising candidates for the next generation of photodetectors. Another promising area for perovskite materials is lasers. Perovskite materials have shown significant progress as laser materials, thanks to their outstanding optical gain, tunable bandgap, and low-cost fabrication processes. Perovskite lasers are expected to be a disruptive technology in areas such as telecommunications, sensing, and medical imaging, offering compact, efficient, and low-cost alternatives to traditional semiconductor lasers. Researchers are focusing on improving the efficiency and operational lifetime of perovskite lasers by addressing challenges such as stability under high-power operation and overcoming the limitations related to perovskite’s sensitivity to environmental factors. Beyond optoelectronics, perovskites have also demonstrated promise in catalysis applications [151]. Perovskite materials, particularly those with mixed metal or mixed anion compositions, exhibit excellent catalytic properties, making them suitable for a wide range of reactions, including water splitting for hydrogen production, CO_2_ reduction, and oxygen evolution reactions (OER).

These reactions are key to advancing clean energy technologies, particularly in the context of sustainable hydrogen production and carbon capture. The tunability of perovskite materials allows for the design of catalysts with high activity and selectivity, providing a pathway for developing efficient and cost-effective catalytic systems for energy conversion and storage. Another exciting application area is ionics and energy storage [152]. Perovskites, particularly in their mixed ion forms, have shown great potential in supercapacitors and batteries, where they can serve as high-capacity electrodes or electrolytes. Their high ionic conductivity and ability to store charge make them excellent candidates for enhancing the performance of energy storage devices [153]. Perovskite-based supercapacitors and batteries could lead to lighter, more efficient, and longer-lasting energy storage solutions. The integration of perovskites into flexible and lightweight energy storage devices is also being explored, which could enable applications in wearable electronics and portable power sources. Furthermore, sensors based on perovskite materials are becoming increasingly popular due to their high sensitivity, low-cost fabrication, and versatility in detecting a variety of substances. Perovskite-based sensors can be used for environmental monitoring, health diagnostics, and industrial applications. Their excellent optoelectronic properties enable them to detect gases, chemicals, and even biological markers with high sensitivity and fast response times [154]. For instance, perovskite-based gas sensors can be used to detect pollutants such as ammonia or carbon monoxide in industrial or urban environments, contributing to improved air quality monitoring and safety. Additionally, thermoelectric is another area where perovskites are being actively explored. Perovskite materials have demonstrated potential as efficient thermoelectric materials for converting waste heat into electricity [155]. This can have applications in power generation, particularly in harvesting heat from industrial processes, vehicles, or electronic devices. The ability to tailor the thermoelectric properties of perovskites through material engineering offers an exciting avenue for improving energy efficiency and contributing to sustainable energy solutions. Finally, flexible electronics is an emerging field where perovskites are gaining attention. Their excellent mechanical flexibility, combined with their optoelectronic properties, makes them ideal candidates for applications in flexible displays, foldable electronics, and wearable devices. The ability to fabricate high-performance perovskite-based devices on flexible substrates paves the way for the development of new consumer electronics that are lightweight, portable, and durable. In conclusion, perovskite materials offer a wide range of applications beyond photovoltaics, including light-emitting devices, photodetectors, lasers, catalysis, energy storage, sensors, thermoelectric, and flexible electronics. Their tunable properties, low-cost fabrication, and versatility make them attractive candidates for a variety of advanced technologies, and ongoing research in these areas is expected to lead to groundbreaking innovations in numerous fields [156].

## 8. Future Directions and Challenges

The future of perovskite solar cells holds immense promise, but several challenges need to be addressed for their widespread commercialization and integration into energy systems (Table 3). One of the primary hurdles is the scalability of production; while laboratory methods have shown high efficiency, transitioning to large-scale manufacturing remains a challenge due to issues with uniformity and process optimization. Ongoing research is focused on developing scalable deposition techniques, such as inkjet printing and slot-die coating, to address these concerns. Another significant challenge is the stability and durability of perovskite materials, which are prone to degradation from moisture, oxygen, and UV light. To overcome this, researchers are working on encapsulation techniques and enhancing material compositions through passivation layers. Additionally, the toxicity of lead in many perovskite materials raises environmental and health concerns, prompting research into lead-free alternatives such as tin- and bismuth-based perovskites. Cost reduction is also crucial, as perovskite solar cells are currently more expensive than silicon-based cells. However, efforts are being made to lower production costs through automation, AI-driven material discovery, and optimizing processing methods. Beyond photovoltaics, perovskites show potential in applications such as energy storage, LEDs, and flexible electronics, making interdisciplinary research crucial to unlocking their full potential. Finally, while perovskite solar cells have demonstrated high efficiency, improving long-term economic viability remains essential, necessitating further advancements in material design, performance consistency, and recycling processes. Addressing these challenges will be key to realizing the widespread adoption of perovskite technologies and their contribution to global energy sustainability.

## 9. Conclusions and Perspectives

In conclusion, perovskite solar cells represent a promising technology that has shown significant advancements in efficiency, cost-effectiveness, and scalability. Their potential to revolutionize the photovoltaic industry, thanks to their ease of processing, the use of earth-abundant materials, and high efficiency, positions them as a key player in the global transition toward renewable energy. However, challenges such as stability, environmental concerns, and scalability need to be addressed for PSCs to become commercially viable and widely adopted. The integration of machine learning, artificial intelligence, and advanced materials discovery is paving the way for further optimization, accelerating the development of new materials and improving the long-term performance of PSCs. Additionally, the versatility of PSCs opens the door for their application in various sectors beyond photovoltaics, such as light-emitting devices, lasers, and sensors. Despite the challenges, the continuous progress in both the fundamental understanding and practical applications of PSCs offers exciting prospects for the future, with the potential to make a significant impact on the global energy landscape and contribute to sustainable development goals

## Figures and Tables

**Table 1:** Characterization techniques and their applications for metal halide perovskites.

Category	Technique	Usage
**Structural**	X-ray Diffraction (XRD)	Determines crystal structure and phase composition
	Raman Spectroscopy (RS)	Identifies molecular vibrations and bonding information
**Morphological**	Scanning Electron Microscopy (SEM)	Examine surface structure and morphology
	Transmission Electron Microscopy (TEM)	Observes the fine structure and nanomorphology
**Optical**	UV-Vis Spectroscopy	Measures optical absorption and transmission
	Photoluminescence (PL)	Assesses light emission properties
	FTIR Spectroscopy	Identifies functional groups and molecular vibrations
**Electrical and Dielectric**	Impedance Spectroscopy (IS)	Analyzes electrical impedance behavior
	Hall Effect Measurements	Measures charge carrier concentration and mobility
	Current–Voltage (I–V) Measurements	Characterizes current–voltage relationship
	TSC Measurements	Analyzes charge carrier dynamics at low temperatures
**Emerging Techniques**	Machine Learning (ML) and AI Integration	Uses data for predictive modeling and optimization

**Table 2:** Comparative analysis of perovskite synthesis methods.

Synthesis Method	Advantages	Limitations	Application Areas
Sol–Gel Method	Low-cost, precise control, scalable	Requires post-annealing, moisture-sensitive	Thin films, bulk ceramics
Spin-Coating	Uniform films, easy process	Solvent-related defects, pinholes	Solar cells, sensors
LARP	Room-temperature processing, tunable nanocrystals	Stability issues, ligand dependency	Quantum dots, LEDs
Thermal Evaporation	Pinhole-free films, solvent-free	High vacuum cost, limited material choice	Photovoltaics, electronics
CVD	Highly uniform, scalable	Complex setup, high cost	High-performance devices
Colloidal Synthesis	Tunable bandgap, high luminescence	Stability concerns, passivation required	LEDs, photodetectors
Template-Assisted Growth	Controlled morphology, high charge mobility	Complex template preparation	Nanostructured solar cells

**Table 3:** Key challenges and future directions in perovskite solar cell development.

Challenge	Current Status	Ongoing Research Efforts	Potential Solutions
Scalability of Production	Small-scale laboratory methods; limited large-scale production	Inkjet printing, slot-die coating, spray deposition	Improve deposition uniformity, optimize processing methods, develop scalable techniques for tandem cells
Stability and Durability	Susceptible to degradation from moisture, oxygen, and UV light	Encapsulation techniques, stability enhancement via passivation layers	Use moisture-resistant coatings, develop lead-free perovskites, improve material compositions
Toxicity and Environmental Impact	Lead-based perovskites pose environmental concerns	Research into lead-free perovskites, safe recycling processes	Replace lead with safer materials like tin or bismuth, develop safe recycling and disposal methods
Cost Reduction	High manufacturing costs in comparison to silicon-based cells	AI-driven material discovery, automation of manufacturing processes	Use machine learning for material optimization, scale production for cost reduction
Integration with Other Technologies	Currently limited to PV applications	Research into hybrid perovskite-based energy storage systems, LEDs, and flexible electronics	Develop integrated systems for solar, storage, and display applications using perovskites
Long-Term Economic Viability	Initial high production cost, limited industrial adoption	Enhancing efficiency, reducing material costs, improving production scalability	Reduce material and processing costs through innovations in material design, improve performance consistency

## Data Availability

Data sharing is not applicable to this article as no datasets were generated or analyzed during the current study.
