# Correction: Tayari et al. Progress and Developments in the Fabrication and Characterization of Metal Halide Perovskites for Photovoltaic Applications. *Nanomaterials* 2025, *15*, 613

**DOI:** 10.3390/nano16050325

**Published:** 2026-03-05

**Authors:** Faouzia Tayari, Silvia Soreto Teixeira, Manuel Pedro F. Graca, Kais Iben Nassar

**Affiliations:** I3N-Aveiro, Department of Physics, University of Aveiro, 3810-193 Aveiro, Portugal; faouzia.tayari@ua.pt (F.T.); silvia.soreto@ua.pt (S.S.T.); mpfg@ua.pt (M.P.F.G.)

In the original publication [1], the citation referring to References [7,8,18,19,22,24,25,29,30,38,44,46,54,57,60,65,68,73,76,79,83,86,89,93,94,102,108,114,118,119,122,126,129,133,148,151,155,162] in the manuscript needed to be revised due to mismatches with the Mendeley reference manager.

Concerns were raised on this matter and References [44,46,54,57,68,79,86,94,114,119,133,151] were removed. Reference [30] was replaced with the original Reference [72]. With this correction, the order of some references has been adjusted accordingly.

The References [7,8,18,19,22,24,25,29,38,60,65,73,76,83,89,93,102,108,118,122,126,129,148,155,162] were replaced with the following references:

7. Liu, X.; Zhao, W.; Cui, H.; Xie, Y.A.; Wang, Y.; Xu, T.; Huang, F. Organic-inorganic halide perovskite based solar cells–revolutionary progress in photovoltaics. *Inorg. Chem. Front.* **2015**, *2*, 315–335. https://doi.org/10.1039/C4QI00163J.
8. Shah, N.; Shah, A.A.; Leung, P.K.; Khan, S.; Sun, K.; Zhu, X.; Liao, Q. A review of third generation solar cells. *Processes* **2023**, *11*, 1852. https://doi.org/10.3390/pr11061852.
18. Machín, A.; Márquez, F. Advancements in photovoltaic cell materials: Silicon, organic, and perovskite solar cells. *Materials* **2024**, *17*, 1165. https://doi.org/10.3390/ma17051165.
19. Koh, T.M.; Wang, H.; Ng, Y.F.; Bruno, A.; Mhaisalkar, S.; Mathews, N. Halide perovskite solar cells for building integrated photovoltaics: Transforming building façades into power generators. *Adv. Mater.* **2022**, *34*, 2104661. https://doi.org/10.1002/adma.202104661.
22. Dipta, S.S.; Rahim, M.A.; Uddin, A. Encapsulating perovskite solar cells for long-term stability and prevention of lead toxicity. *Appl. Phys. Rev.* **2024**, *11*, 021301. https://doi.org/10.1063/5.0197154.
24. Tailor, N.K.; Abdi-Jalebi, M.; Gupta, V.; Hu, H.; Dar, M.I.; Li, G.; Satapathi, S. Recent progress in morphology optimization in perovskite solar cell. *J. Mater. Chem. A* **2020**, *8*, 21356–21386. https://doi.org/10.1039/D0TA00143K.
25. Salim, T.; Sun, S.; Abe, Y.; Krishna, A.; Grimsdale, A.C.; Lam, Y.M. Perovskite-based solar cells: Impact of morphology and device architecture on device performance. *J. Mater. Chem. A* **2015**, *3*, 8943–8969. https://doi.org/10.1039/C4TA05226A.
29. Tirmzi, A.M.; Dwyer, R.P.; Jiang, F.; Marohn, J.A. Light-dependent impedance spectra and transient photoconductivity in a ruddlesden-popper 2D lead-halide perovskite revealed by electrical scanned probe microscopy and accompanying theory. *J. Phys. Chem. C* **2020**, *124*, 13639–13648. https://doi.org/10.1021/acs.jpcc.0c04467.
38. Abdulrahim, S.M.; Ahmad, Z.; Bahadra, J.; Al-Thani, N.J. Electrochemical impedance spectroscopy analysis of hole transporting material free mesoporous and planar perovskite solar cells. *Nanomaterials* **2020**, *10*, 1635. https://doi.org/10.3390/nano10091635.
60. Ibaceta-Jaña, J.; Muydinov, R.; Rosado, P.; Mirhosseini, H.; Chugh, M.; Nazarenko, O.; Hoffmann, A. Vibrational dynamics in lead halide hybrid perovskites investigated by Raman spectroscopy. *Phys. Chem. Chem. Phys.* **2020**, *22*, 5604–5614. https://doi.org/10.1039/C9CP06568G.
65. Shih, M.C.; Li, S.S.; Hsieh, C.H.; Wang, Y.C.; Yang, H.D.; Chiu, Y.P.; Chen, C.W. Spatially resolved imaging on photocarrier generations and band alignments at perovskite/PbI_2_ heterointerfaces of perovskite solar cells by light-modulated scanning tunneling microscopy. *Nano Lett.* **2017**, *17*, 1154–1160. https://doi.org/10.1021/acs.nanolett.6b04803.
73. Anjum, D.H. Characterization of nanomaterials with transmission electron microscopy. *IOP Conf. Ser. Mater. Sci. Eng.* **2016**, *146*, 012001. https://doi.org/10.1088/1757-899X/146/1/012001.
76. Bao, L.; Gao, P.; Song, T.; Xu, F.; Li, Z.; Xu, G. Atomic-scale imaging of organic-inorganic hybrid perovskite using transmission electron microscope. *Crystals* **2023**, *13*, 973. https://doi.org/10.3390/cryst13060973.
83. Zhang, J.; Yan, S.; Liu, Z.; Ma, N.; Liu, M.; Gao, Y.; Tang, J. Understanding perovskite light-emitting diodes through transmission electron microscopy: Materials structure, optical regulation and devices. *Nano Eng.* **2025**, *135*, 110627. https://doi.org/10.1016/j.nanoen.2024.110627.
89. Chen, S.; Gao, P. Challenges, myths, and opportunities of electron microscopy on halide perovskites. *J. Appl. Phys.* **2020**, *128*, 010901. https://doi.org/10.1063/5.0012310.
93. Li, G.; Wang, Y.; Huang, L.; Zhang, X.; Yang, J.; Qiu, X.; Sun, W. High-performance UV–Vis–NIR photodetectors based on perovskite/PDPP3T polymer composites. *Ceram. Int.* **2023**, *49*, 13860–13871. https://doi.org/10.1016/j.ceramint.2022.12.266.
102. Seth, S.; Samanta, A. Photoluminescence of zero-dimensional perovskites and perovskite-related materials. *J. Phys. Chem. Lett.* **2018**, *9*, 176–183. https://doi.org/10.1021/acs.jpclett.7b02931.
108. Li, J.; Yuan, X.; Jing, P.; Li, J.; Wei, M.; Hua, J.; Tian, L. Temperature-dependent photoluminescence of inorganic perovskite nanocrystal films. *RSC Adv.* **2016**, *6*, 78311–78316. https://doi.org/10.1039/C6RA17008K.
118. Li, S.Y.; Fan, R.; Chen, X.H.; Wang, C.H.; Mo, W.Q.; Ruan, K.Q.; Cao, L.Z. Normal state resistivity, upper critical field, and Hall effect in superconducting perovskite MgCNi_3_. *Phys. Rev. B* **2001**, *64*, 132505. https://doi.org/10.1103/PhysRevB.64.132505.
122. Unger, E.L.; Hoke, E.T.; Bailie, C.D.; Nguyen, W.H.; Bowring, A.R.; Heumüller, T.; McGehee, M.D. Hysteresis and transient behavior in current–voltage measurements of hybrid-perovskite absorber solar cells. *Energy Environ. Sci.* **2014**, *7*, 3690–3698. https://doi.org/10.1039/C4EE02465F.
126. Butt, M.A. Thin-film coating methods: A successful marriage of high-quality and cost-effectiveness—A brief exploration. *Coatings* **2022**, *12*, 1115. https://doi.org/10.3390/coatings12081115.
129. Lee, J.H.; Kim, B.S.; Park, J.; Lee, J.W.; Kim, K. Opportunities and challenges for perovskite solar cells based on vacuum thermal evaporation. *Adv. Mater. Technol.* **2023**, *8*, 2200928. https://doi.org/10.1002/admt.202200928.
148. Tong, X.; Lin, F.; Wu, J.; Wang, Z.M. High performance perovskite solar cells. *Adv. Sci.* **2016**, *3*, 1500201. https://doi.org/10.1002/advs.201500201.
155. Xia, J.; Sohail, M.; Nazeeruddin, M.K. Efficient and stable perovskite solar cells by tailoring of interfaces. *Adv. Mater.* **2023**, *35*, 2211324. https://doi.org/10.1002/adma.202211324.
162. Kim, Y.H.; Kim, J.S.; Lee, T.W. Strategies to improve luminescence efficiency of metal-halide perovskites and light-emitting diodes. *Adv. Mater.* **2019**, *31*, 1804595. https://doi.org/10.1002/adma.201804595.

The authors confirm that these changes do not affect the content, context, interpretation, or conclusions of the article in any way. All remaining references have been verified manually for accuracy, relevance, and proper citation. This correction was approved by the Academic Editor. The original publication has also been updated.
